# Virtual Sensing of Unmeasured Supply–Return Disturbances for Predictive Control in Cooperative Hydraulic Support Pushing

**DOI:** 10.3390/s26144363

**Published:** 2026-07-09

**Authors:** Tiangu Wu, Lijuan Zhao, Jiazheng Bu, Zhanpeng Zhang, Beichen Jiang, Guocong Lin, Shutian Gong

**Affiliations:** 1 School of Mechanical Engineering, Liaoning Technical University, Fuxin 123000, China; 2 Liaoning Provincial Technical Innovation Center for Hydraulic Transmission and Control, Fuxin 123000, China

**Keywords:** virtual sensing, sensor-limited control, pressure-boundary estimation, disturbance reconstruction, hydraulic support, predictive control

## Abstract

Cooperative pushing of hydraulic supports is affected by pressure-boundary fluctuations and neighboring-branch actions transmitted through shared supply–return circuits. In longwall mining systems, common-node pressures and neighboring-branch flow disturbances are difficult to measure continuously, which limits their use in feedback predictive control. This study proposes a virtual-sensing-based output -feedback predictive control method for cooperative hydraulic support pushing under limited sensing conditions. A control-oriented coupled model is established by retaining the two-chamber pressure dynamics of the target cylinder, supply-main impedance, common return-main dynamics, and equivalent neighboring supply-side and return-side disturbance flows. The equivalent disturbance flows are introduced as nodal disturbance inputs in the supply and return pressure dynamics. A linear extended state observer reconstructs the common supply-node pressure, common return-node pressure, and equivalent neighboring-disturbance flows from target-cylinder measurements, including displacement, velocity, cap-end pressure, and rod-end pressure. The reconstructed variables are incorporated into an output-feedback model predictive controller to coordinate displacement tracking, velocity stabilization, pressure-boundary regulation, and valve-input smoothing under input-amplitude, input-increment, and output constraints. The method is validated using AMESim–MATLAB/Simulink co-simulation and a dual-branch hydraulic experimental platform. Co-simulation results show that the proposed controller gives a maximum velocity deviation of 1.7484 mm/s, a recovery time of 0.112 s within the 1% velocity error band, and an input total variation of 2.3383. Experimental results show that controller intervention reduces the maximum velocity fluctuation from 1.0041 mm/s to 0.3011 mm/s and shortens the recovery time from 0.505 s to 0.038 s. The results demonstrate that virtual sensing of supply–return pressure boundaries and equivalent neighboring-disturbance flows improves motion continuity and driving-pressure stability in cooperative hydraulic actuation under limited sensing conditions.

## 1. Introduction

With the rapid development of intelligent coal mining, hydraulic supports in fully mechanized longwall faces are evolving from isolated actuation units to support groups capable of cooperative perception, shearer-following advancement, and intelligent control. Cooperative pushing of hydraulic supports is not a simple single-cylinder displacement-tracking problem. Instead, it is a coupled process involving the hydraulic support, scraper conveyor, common supply–return circuits, neighboring support action sequences, and closed-loop controller execution. In particular, when multiple supports share the same supply and return mains, neighboring support start–stop operations, rapid valve closure, or large-flow actions can induce fluctuations in the common supply pressure and return backpressure. These fluctuations further modify the pressure difference between the two chambers of the target support cylinder, the valve-orifice flow rates, and the pushing velocity. Therefore, the key challenge in cooperative pushing control is not only to track the target-cylinder displacement, but also to suppress velocity fluctuations, maintain stable pressure boundaries, and ensure smooth valve-control inputs under shared supply–return disturbances. For cooperative pushing under shared hydraulic circuits, the main disturbance source is associated with pressure-boundary variation rather than only mechanical-side load fluctuation. When a neighboring support starts, stops, or closes its valve rapidly, the corresponding change in flow demand modifies the common supply pressure and return backpressure. These boundary variations enter the target-cylinder dynamics through the valve-orifice pressure differences and then affect the pushing velocity. Therefore, the control problem requires the reconstruction and regulation of unmeasured supply–return pressure boundaries in addition to local displacement and chamber-pressure feedback.

Substantial research has been conducted on intelligent perception, group cooperative control, and shearer-following decision-making for hydraulic supports. Zhao et al. [1] systematically reviewed the development status and intelligent trends of high-power supply systems for hydraulic supports, emphasizing that reliable supply and dynamic pressure coordination are essential for intelligent longwall equipment. Lian et al. [2] proposed a networked intelligent sensing and control method for hydraulic supports, providing a technical basis for support-state perception, motion-process recognition, and intelligent control. Fu et al. [3] developed an intelligent decision-making model for shearer-following advancement of hydraulic support groups, enabling optimized decisions on support action sequences and pushing velocity. Further, Fu et al. [4] proposed a multimodal human–machine cooperative control system for hydraulic support following under complex working-face conditions, improving the adaptability of group action decisions. Jiao et al. [5] investigated a digital-twin-based decision method for pose self-adjustment of hydraulic support groups, while Li et al. [6] used digital-twin technology for virtual commissioning of the shearer-following process. These studies have promoted the transition of hydraulic supports from single-machine automatic control to group-level intelligent coordination. However, most of them focus on support posture, action sequence, shearer-following decision-making, and virtual commissioning, while the propagation of pressure disturbances in common supply–return mains and its influence on the local motion continuity of the target support remain insufficiently addressed.

For hydraulic actuation systems, model predictive control has been widely adopted because it can explicitly handle multivariable coupling, input saturation, state constraints, and receding-horizon optimization. Gu et al. [7] proposed an output-feedback model predictive control method with disturbance compensation for hydraulic systems, providing an important reference for predictive control under incomplete state measurement. Cho et al. [8] applied model predictive control to a pump-controlled hydraulic system for a legged robot and achieved effective performance under supply-pressure constraints and energy-optimization requirements. Li et al. [9] combined nonlinear model predictive control with cross-coupled control for synchronization of multiple hydraulic systems, improving the synchronization accuracy of multiple hydraulic actuators. Helian et al. [10] studied motion control of electro-hydraulic actuators under multiple time-varying constraints, demonstrating the importance of explicit constraint handling in high-performance hydraulic actuation. Overall, MPC provides a suitable control framework for constrained mechanical–hydraulic coupled systems such as cooperative pushing of hydraulic supports. However, conventional MPC generally relies on relatively complete state measurement or accurate boundary-pressure inputs. In practical longwall faces, the common supply-node pressure, common return-node pressure, and neighboring supply–return disturbances are often difficult to measure online, which degrades prediction accuracy and limits engineering implementation.

To address unknown disturbances and unmeasured states in hydraulic systems, disturbance observers, extended state observers, and output-feedback control methods have become important research directions. Chen et al. [11] reviewed disturbance-observer-based control methods and pointed out that estimating and compensating for system uncertainties and external disturbances through observer mechanisms is an effective way to improve the robustness of complex industrial systems. Shen et al. [12] designed an extended-state-observer-based event-triggered controller for hydraulic position tracking systems to reduce communication and control-update burden. Liang et al. [13] proposed an extended-state-observer-assisted adaptive dynamic-surface prescribed-performance controller for electro-hydraulic actuators subject to parameter uncertainties and matched/unmatched disturbances, achieving effective disturbance rejection. Mayne [14] emphasized that MPC can explicitly handle multivariable constraints and perform receding-horizon optimization over a finite horizon, which provides a theoretical basis for combining state observation with predictive control. Existing studies show that integrating observers with MPC can alleviate unmeasured-state and unknown-disturbance problems to some extent. Nevertheless, most existing applications still focus on single actuators or local hydraulic systems, where the observed variables are usually actuator velocity, load disturbance, or lumped uncertainty. The reconstruction of common pressure boundaries and neighboring flow disturbances in shared supply–return networks has not been fully considered. From the viewpoint of predictive control, missing common-node pressures and neighboring-branch disturbances lead to incomplete boundary information in the prediction model. If these variables are treated only as lumped uncertainties, the controller can compensate for their resultant effect but cannot explicitly describe how supply-side and return-side disturbances modify the inlet and outlet pressure differences in the target cylinder. For this reason, this study introduces the common supply-node pressure, common return-node pressure, and equivalent neighboring supply-side and return-side disturbance flows as structured interface variables between virtual sensing and predictive control.

In cooperative pushing of hydraulic supports, the common supply-node pressure $P_{s}$, common return-node pressure $P_{r}$, and equivalent neighboring supply–return disturbances $q_{n,s}$ and $q_{n,r}$ are critical variables affecting the velocity and pressure responses of the target cylinder. On the supply side, rapid valve closure of a neighboring support reduces the instantaneous flow demand in the common supply circuit, leading to a transient rise in the supply-node pressure. On the return side, changes in the discharge state of the neighboring support propagate through the common return main, modifying the return backpressure near the target branch and changing the discharge pressure difference in the rod chamber. These supply-side and return-side disturbances jointly act on the valve-orifice pressure differences in the target cylinder and induce transient fluctuations in pushing velocity. In practical applications, installing high-frequency pressure and flow sensors at every common node and neighboring branch would increase cost and installation complexity, while sensor reliability and maintainability would also be limited by the harsh underground environment. Recent advances in digital twins, virtual testing, and support-state assessment have provided new routes to reducing dependence on field measurements [15,16,17]. It is therefore necessary to reconstruct the common supply–return boundaries and neighboring disturbances online from measurable target-cylinder information, including displacement, velocity, and two chamber pressures, and further embed the reconstructed information into a predictive controller for output-feedback control.

Based on the above analysis, this paper proposes a virtual-sensing-based output-feedback model predictive control method for cooperative hydraulic support pushing under shared supply–return disturbances. A control-oriented coupled model is first formulated by retaining the dominant channels through which neighboring actions affect the target cylinder, including the supply-main impedance, common return-main dynamics, and equivalent nodal disturbance flows. A linear extended state observer is then constructed to reconstruct the common supply-node pressure, common return-node pressure, and equivalent neighboring-disturbance flows from the target-cylinder displacement, velocity, cap-end pressure, and rod-end pressure. The reconstructed variables are incorporated into the prediction model of an output-feedback MPC, where displacement tracking, velocity stabilization, pressure-boundary regulation, and valve-input smoothing are optimized under practical constraints. The method is validated through AMESim2020 and MATLAB2025b/Simulink co-simulation and a dual-branch hydraulic experimental platform.

The main contributions of this paper are summarized as follows.

(1) A control-oriented shared supply–return coupling model is established for cooperative hydraulic support pushing. The model retains the target-cylinder pressure dynamics, supply-main impedance, common return-main dynamics, and equivalent neighboring supply-side and return-side disturbance flows. This formulation provides a tractable model interface for describing how neighboring support actions modify the pressure boundaries of the target cylinder.
(2) A virtual sensing strategy is developed for reconstructing unmeasured supply–return boundary variables under limited sensing conditions. The common supply-node pressure, common return-node pressure, and equivalent neighboring-disturbance flows are estimated from target-cylinder measurements and are organized as controller-interface variables for subsequent predictive control.
(3) An observer-based output-feedback MPC is designed by embedding the reconstructed boundary and disturbance variables into the prediction model. The controller coordinates displacement tracking, velocity stabilization, pressure-boundary regulation, and valve-input smoothing under input-amplitude, input-increment, and output constraints.
(4) The proposed method is evaluated using co-simulation and hydraulic-platform experiments. The validation includes observer reconstruction, comparative control performance, disturbance-response analysis, and implementation-oriented evaluation of robustness and computational feasibility.

The remainder of this paper is organized as follows. Section 2 establishes the simplified coupled model for cooperative pushing of hydraulic supports under dual supply–return disturbances. Section 3 designs the extended state observer for unmeasured-state reconstruction. Section 4 develops the observer-based output-feedback model predictive controller. Section 5 validates the proposed method through co-simulation and hydraulic-platform experiments. Section 6 concludes the paper.

## 2. Simplified Multi-Coupling Model for Cooperative Pushing of Hydraulic Supports

To support unmeasured-state reconstruction and output-feedback predictive controller design, a control-oriented coupled model is established for cooperative pushing of hydraulic supports. The model retains the target pushing cylinder, the impedance of the supply main, and the dynamics of the common return main. The effects of neighboring support start–stop operations and valve closure on the supply–return boundaries of the target support are represented as external equivalent flow disturbances [18]. This formulation describes the dominant coupling channels required for observer construction and predictive controller synthesis.

The disturbance flow induced by neighboring support actions is denoted as:

(1) $$q_{n}={\left[\begin{array}{cc} q_{n,s} & q_{n,r} \end{array}\right]}^{T}$$

where $q_{n,s}$ acts on the common supply node and represents the variation in supply-side flow demand caused by neighboring support start–stop operations or valve closure; $q_{n,r}$ acts on the common return node and represents the return-side backpressure disturbance caused by changes in the discharge flow of the neighboring support.

### 2.1. Hydraulic Cylinder–Load Subsystem Dynamics

The target hydraulic cylinder drives the pushing mechanism under the pressure difference between the two chambers. The mechanical-side dynamics are expressed as:

(2) $$M\dot{v}=A_{a}P_{a}-A_{b}P_{b}-Bv-F_{d}$$

where M is the equivalent moving mass; B is the equivalent viscous damping coefficient; $A_{a}$ and $A_{b}$ are the effective areas of the cap-end and rod-end chambers of the hydraulic cylinder, respectively; and $F_{d}$ is the lumped external load on the mechanical side.

The displacement–velocity relation is given by:

(3) $$\dot{x}=v$$

According to the flow continuity of the two cylinder chambers, the pressure dynamics of the cap-end and rod-end chambers are respectively written as:

(4) $${\dot{P}}_{a}=\frac{\beta_{e}}{V_{a0}+A_{a}x}\left(q_{a}-A_{a}v-C_{t}(P_{a}-P_{b})\right)$$

(5) $${\dot{P}}_{b}=\frac{\beta_{e}}{V_{b0}-A_{b}x}\left(A_{b}v-q_{b}+C_{t}(P_{a}-P_{b})\right)$$

where $\beta_{e}$ is the effective bulk modulus of the emulsion; $V_{a0}$ and $V_{b0}$ are the initial control volumes of the two cylinder chambers; $C_{t}$ is the equivalent internal leakage coefficient; and $q_{a}$ and $q_{b}$ are the valve-orifice flow rates entering the cap-end chamber and leaving the rod-end chamber, respectively.

For the pushing extension condition, the valve-orifice flow rates are expressed as:

(6) $$q_{a}=C_{d}wu\sqrt{\frac{2}{\rho }(P_{s}-P_{a})}$$

(7) $$q_{b}=C_{d}wu\sqrt{\frac{2}{\rho }(P_{b}-P_{r})}$$

where $C_{d}$ is the discharge coefficient, w is the valve-orifice area gradient, u is the equivalent spool control input, and ρ is the density of the emulsion.

Equations (6) and (7) indicate that the common supply-node pressure $P_{s}$ and the common return-node pressure $P_{r}$ affect the velocity response of the target hydraulic cylinder through the inlet and outlet pressure differences, respectively.

### 2.2. Supply-Side Coupling Dynamics

Considering the pipeline impedance between the pump station and the supply node of the target support, the supply main is represented as a hydraulic resistance–hydraulic inertance element:

(8) $$L_{s}{\dot{q}}_{s}=P_{0}-P_{s}-R_{s}q_{s}$$

where $L_{s}$ and $R_{s}$ are the equivalent hydraulic inertance and hydraulic resistance of the supply main, respectively, and $P_{0}$ is the equivalent supply pressure at the remote pump station.

According to the flow balance at the supply node, the supply-node pressure dynamics are obtained as:

(9) $${\dot{P}}_{s}=\frac{\beta_{e}}{V_{s}}\left(q_{s}+q_{n,s}-q_{a}\right)$$

where $V_{s}$ is the equivalent control volume of the supply node, and $q_{n,s}$ is the equivalent supply-side disturbance flow induced by neighboring support actions. In the proposed model, $q_{n,s}$ acts as an equivalent nodal disturbance input in the supply-node continuity equation. When the neighboring valve closes rapidly, the instantaneous supply-side flow demand decreases, which can be represented by $q_{n,s}>0$ and causes a transient rise in $P_{s}$. Conversely, when the neighboring support opens rapidly or draws a large flow rate, it can be represented by $q_{n,s}<0$, leading to a transient drop in $P_{s}$ [19].

### 2.3. Common Return-Side Disturbance Model

The oil discharge from the rod-end chamber of the target support is governed not only by its own valve opening but also by the common return-node pressure $P_{r}$ [20]. The pressure dynamics of the common return node are written as:

(10) $${\dot{P}}_{r}=\frac{\beta_{r}}{V_{r}}\left(q_{b}+q_{n,r}-q_{r}\right)$$

where $\beta_{r}$ is the equivalent bulk modulus of the gas–liquid mixture in the common return main, $V_{r}$ is the equivalent control volume of the common return node, and $q_{n,r}$ is the equivalent return-side disturbance flow induced by neighboring support actions. Similarly, $q_{n,r}$ acts as an equivalent nodal disturbance input in the return-node continuity equation.

The flow dynamics of the common return main are described by:

(11) $$L_{r}{\dot{q}}_{r}=P_{r}-P_{t}-R_{r}q_{r}$$

where $L_{r}$ and $R_{r}$ are the equivalent hydraulic inertance and hydraulic resistance of the common return main, respectively, and $P_{t}$ is the equivalent tank return pressure.

Equations (10) and (11) show that changes in the neighboring discharge flow can modify $P_{r}$ through the common return main. This variation then affects the rod-end discharge flow and the motion response of the target hydraulic cylinder through Equation (7).

### 2.4. Simplified State-Space Model

For observer design and output-feedback controller synthesis, the system state vector is defined as:

(12) $$X={\left[\begin{array}{cccccccc} x & v & P_{a} & P_{b} & P_{s} & q_{s} & P_{r} & q_{r} \end{array}\right]}^{T}$$

The control input is u, and the external disturbance vector is defined as:

(13) $$\omega ={\left[\begin{array}{cc} q_{n,s} & q_{n,r} \end{array}\right]}^{T}$$

The total external disturbance vector is further defined as:

(14) $$D={\left[\begin{array}{cc} F_{d} & \omega^{T} \end{array}\right]}^{T}$$

Accordingly, the system can be written in the following compact form:

(15) $$\dot{X}=f(X,u,D)$$

The specific state equations are given by:

(16) $$\left\{\begin{array}{l} \dot{x}=v\\ M\dot{v}=A_{a}P_{a}-A_{b}P_{b}-Bv-F_{d}\\ {\dot{P}}_{a}=\frac{\beta_{e}}{V_{a0}+A_{a}x}\left[q_{a}-A_{a}v-C_{t}(P_{a}-P_{b})\right]\\ {\dot{P}}_{b}=\frac{\beta_{e}}{V_{b0}-A_{b}x}\left[A_{b}v-q_{b}+C_{t}(P_{a}-P_{b})\right]\\ {\dot{P}}_{s}=\frac{\beta_{e}}{V_{s}}\left(q_{s}+q_{n,s}-q_{a}\right)\\ L_{s}{\dot{q}}_{s}=P_{0}-P_{s}-R_{s}q_{s}\\ {\dot{P}}_{r}=\frac{\beta_{r}}{V_{r}}\left(q_{b}+q_{n,r}-q_{r}\right)\\ L_{r}{\dot{q}}_{r}=P_{r}-P_{t}-R_{r}q_{r} \end{array}\right.$$

The corresponding valve-orifice flow equations are:

(17) $$q_{a}=C_{d}wu\sqrt{\frac{2}{\rho }(P_{s}-P_{a})}$$

(18) $$q_{b}=C_{d}wu\sqrt{\frac{2}{\rho }(P_{b}-P_{r})}$$

Equations (15)–(18) constitute a control-oriented nonlinear model of the target pushing cylinder under supply–return disturbances. In this model, $q_{n,s}$ describes the influence of neighboring support actions on the supply-node pressure, while $q_{n,r}$ describes the influence of neighboring discharge-flow variations on the common return backpressure. Without adding a detailed model of the neighboring actuator, the proposed formulation retains the dominant coupling channels through which supply-side and return-side disturbances affect the motion response of the target support. The variables $q_{n,s}$ and $q_{n,r}$ should be interpreted within this control-oriented model as equivalent inputs that summarize the influence of neighboring actions on the local supply and return pressure dynamics.

### 2.5. Modeling Scope

The model in Section 2.1, Section 2.2, Section 2.3 and Section 2.4 is used as the observation and prediction model for the target branch. The target pushing cylinder is considered under the extension condition, the valve-orifice flows are described by quasi-steady orifice equations, and the supply and return mains are represented by lumped resistance, inertance, and control-volume dynamics. Neighboring support actions enter the supply-node and return-node continuity equations through the equivalent disturbance inputs $q_{n,s}$ and $q_{n,r}$. The mechanical load variation and model residuals are treated as bounded external terms.

The model is applicable to cooperative pushing conditions in which the coupling between the target branch and neighboring branches is mainly governed by shared supply and return pressure boundaries. The considered operating region involves bounded pressure fluctuations and finite valve-opening variations. Strong water-hammer transients, sustained cavitation, severe aeration, valve dead-zone discontinuities, and high-order distributed pipeline effects are outside the model scope and may appear as prediction errors or transient observation errors in the subsequent control process.

## 3. Extended State Observer Design for Unmeasured-State Reconstruction

As shown in Section 2, the motion of the target hydraulic cylinder is affected not only by its own valve input and chamber pressures, but also by the common supply-node pressure $P_{s}$, common return-node pressure $P_{r}$, and equivalent neighboring supply–return disturbances $q_{n,s}$ and $q_{n,r}$. In practical engineering applications, the displacement, velocity, and two chamber pressures of the target hydraulic cylinder can usually be obtained using displacement sensors, differential filtering, and pressure sensors. However, the common supply–return-node pressures and neighboring disturbances are difficult to measure directly. Therefore, based on the supply–return disturbance coupling model established in Section 2, an extended state observer is designed to reconstruct the common pressure boundaries and neighboring supply–return disturbances online, and to provide state-completion information for the subsequent output-feedback MPC.

### 3.1. Observation Problem Description and State Decomposition

According to the definition in Section 2, the system state vector can be written as:

(19) $$X={\left[\begin{array}{cccccccc} x & v & P_{a} & P_{b} & P_{s} & q_{s} & P_{r} & q_{r} \end{array}\right]}^{T}$$

Considering field measurement conditions, the measurable output is defined as the pushing displacement, velocity, cap-end chamber pressure, and rod-end chamber pressure [21], namely:

(20) $$Y={\left[\begin{array}{cccc} x & v & P_{a} & P_{b} \end{array}\right]}^{T}$$

where the output matrix is given by:

(21) $$C=\left[\begin{array}{cccccccc} 1 & 0 & 0 & 0 & 0 & 0 & 0 & 0\\ 0 & 1 & 0 & 0 & 0 & 0 & 0 & 0\\ 0 & 0 & 1 & 0 & 0 & 0 & 0 & 0\\ 0 & 0 & 0 & 1 & 0 & 0 & 0 & 0 \end{array}\right]$$

The equivalent supply-side and return-side disturbances induced by neighboring support actions are defined as:

(22) $$\omega ={\left[\begin{array}{cc} q_{n,s} & q_{n,r} \end{array}\right]}^{T}$$

where $q_{n,s}$ denotes the equivalent disturbance acting on the common supply node caused by neighboring support start–stop operations or valve closure, and $q_{n,r}$ denotes the equivalent disturbance acting on the common return node caused by changes in the neighboring discharge flow.

According to the available measurements, the unmeasured internal-state subset in X and the set of common pressure boundaries and neighboring disturbances called by the output-feedback MPC are respectively defined as:

(23) $$\begin{array}{l} X_{u}={\left[\begin{array}{cccc} P_{s} & P_{r} & q_{s} & q_{r} \end{array}\right]}^{T}\\ X_{c}={\left[\begin{array}{cccc} P_{s} & P_{r} & q_{n,s} & q_{n,r} \end{array}\right]}^{T} \end{array}$$

where $X_{u}$ is the subset of internal states in X that cannot be directly measured. $X_{c}$ is the controller-interface variable. In $X_{c}$, $P_{s}$ and $P_{r}$ are taken from the system state X, while $q_{n,s}$ and $q_{n,r}$ are taken from the disturbance vector ω. Therefore, $X_{c}$ is not a new independent state vector, but a combination of boundary states and disturbance variables directly used by the subsequent output-feedback MPC. This definition gives a direct interface between the observer and the controller, where the estimated common-node pressures and equivalent nodal disturbance flows are supplied to the prediction model at each sampling instant.

### 3.2. Operating-Point Linearization and Augmented Model Construction

The nonlinear plant model in Section 2 can be uniformly written as:

(24) $$\dot{X}=f(X,u,\omega ,F_{d}),\;Y=h(X)$$

At each sampling instant, a nominal operating point is selected as:

(25) $$\bar{X}={\left[\begin{array}{cccccccc} \bar{x} & \bar{v} & {\bar{P}}_{a} & {\bar{P}}_{b} & {\bar{P}}_{s} & {\bar{q}}_{s} & {\bar{P}}_{r} & {\bar{q}}_{r} \end{array}\right]}^{T}$$

The deviations of the variables from the operating point are defined as:

(26) $$\Delta X=X-\bar{X},\;\Delta u=u-\bar{u},\;\Delta \omega =\omega -\bar{\omega },\;\Delta F_{d}=F_{d}-{\bar{F}}_{d}$$

By applying a first-order Taylor expansion to Equation (24) around the current operating point and neglecting higher-order terms, the following local linear deviation model is obtained:

(27) $$\Delta \dot{X}=A\Delta X+B\Delta u+E\Delta \omega +G\Delta F_{d}$$

The output deviation equation is:

(28) ΔY=CΔX

where the matrices are obtained from the Jacobian matrices of the nonlinear model evaluated at the nominal operating point:

(29) $$A={\left.\frac{\partial f}{\partial X}\right|}_{\bar{X},\bar{u},\bar{\omega },{\bar{F}}_{d}},\;B={\left.\frac{\partial f}{\partial u}\right|}_{\bar{X},\bar{u},\bar{\omega },{\bar{F}}_{d}},\;E={\left.\frac{\partial f}{\partial \omega }\right|}_{\bar{X},\bar{u},\bar{\omega },{\bar{F}}_{d}},\;G={\left.\frac{\partial f}{\partial F_{d}}\right|}_{\bar{X},\bar{u},\bar{\omega },{\bar{F}}_{d}}$$

The equivalent neighboring disturbance is generally bounded within a single sampling period. Its variation is defined as:

(30) $$\Delta \dot{\omega }=\nu ,\;\|\nu \|\leq \bar{\nu }$$

where ν is the rate of change in the equivalent neighboring disturbance, and $\bar{\nu }$ is its upper bound. To simultaneously estimate the system-state deviation and the neighboring-disturbance deviation, the augmented state is defined as:

(31) $$\xi ={\left[\begin{array}{cc} \Delta X^{T} & \Delta \omega^{T} \end{array}\right]}^{T}$$

The system can then be rewritten as:

(32) $$\dot{\xi }=A_{a}\xi +B_{a}\Delta u+G_{a}\Delta F_{d}+E_{a}\nu$$

The corresponding output equation is:

(33) $$\Delta Y=C_{a}\xi$$

where the augmented matrices are defined as:

(34) $$A_{a}=\left[\begin{array}{cc} A & E\\ 0 & 0 \end{array}\right],\;B_{a}=\left[\begin{array}{c} B\\ 0 \end{array}\right],\;G_{a}=\left[\begin{array}{c} G\\ 0 \end{array}\right],\;E_{a}=\left[\begin{array}{c} 0\\ I \end{array}\right],\;C_{a}=\left[\begin{array}{cc} C & 0 \end{array}\right]$$

Equations (31)–(34) transform the reconstruction of common supply–return pressure boundaries and equivalent neighboring disturbances into an augmented-state observation problem. Since ΔX contains $\Delta P_{s}$ and $\Delta P_{r}$, and Δω contains $\Delta q_{n,s}$ and $\Delta q_{n,r}$, the common boundaries and disturbance estimates required by the controller can be obtained by observing the augmented state ξ.

### 3.3. Linear Extended State Observer Design

For the augmented model described by Equations (32) and (33), a linear extended state observer is constructed as:

(35) $$\dot{\hat{\xi }}=A_{a}\hat{\xi }+B_{a}\Delta u+L\left(\Delta Y-\Delta \hat{Y}\right)$$

where the estimated output is:

(36) $$\Delta \hat{Y}=C_{a}\hat{\xi }$$

Here, $\hat{\xi }$ is the estimate of the augmented state, and L is the observer gain matrix. Expanding $\hat{\xi }$ gives:

(37) $$\hat{\xi }={\left[\begin{array}{cc} \Delta {\hat{X}}^{T} & \Delta {\hat{\omega }}^{T} \end{array}\right]}^{T}$$

The estimates of the system state and the equivalent neighboring disturbance are therefore obtained as:

(38) $$\hat{X}=\bar{X}+\Delta \hat{X},\;\hat{\omega }=\bar{\omega }+\Delta \hat{\omega }$$

The common pressure boundaries and neighboring disturbances used by the controller are then reconstructed as:

(39) $${\hat{X}}_{c}={\left[\begin{array}{cccc} {\hat{P}}_{s} & {\hat{P}}_{r} & {\hat{q}}_{n,s} & {\hat{q}}_{n,r} \end{array}\right]}^{T}$$

where ${\hat{P}}_{s}$ and ${\hat{P}}_{r}$ are obtained from the corresponding common supply-node pressure and common return-node pressure components in $\hat{X}$; ${\hat{q}}_{n,s}$ and ${\hat{q}}_{n,r}$ are obtained from the corresponding supply-side and return-side equivalent disturbance components in $\hat{\omega }$.

In the observer, the model term $A_{a}\hat{\xi }+B_{a}\Delta u$ predicts the augmented state, while the output-error correction term $L(\Delta Y-\Delta \hat{Y})$ corrects the estimation error using the measurable target-cylinder-side variables x, v, $P_{a}$, and $P_{b}$. The observer gain L can be selected by pole placement so that $A_{a}-LC_{a}$ is a Hurwitz matrix. To balance convergence speed and noise robustness, the observer poles should be faster than the dominant closed-loop poles, but should not be placed excessively far to the left, since this would amplify velocity-differentiation noise and pressure-measurement noise.

The reconstruction is performed under the augmented model defined in Equations (31)–(34). For a fixed operating point, the observer gain is selected after checking the detectability of $\left(A_{a},C_{a}\right)$. The estimated disturbance components therefore correspond to the equivalent disturbance inputs in the adopted augmented model.

### 3.4. Boundedness Analysis of the Observation Error

The augmented-state estimation error is defined as [22]:

(40) $$e=\xi -\hat{\xi }$$

Subtracting the observer equation from the augmented model yields the error dynamics:

(41) $$\dot{e}=(A_{a}-LC_{a})e+G_{a}\Delta F_{d}+E_{a}\nu$$

The effects of the mechanical-side load deviation and the neighboring-disturbance variation rate on the observation error are combined as:

(42) $$d_{o}=G_{a}\Delta F_{d}+E_{a}\nu ,\;\|d_{o}\|\leq {\bar{d}}_{o}$$

Thus, the error dynamics can be rewritten as:

(43) $$\dot{e}=(A_{a}-LC_{a})e+d_{o}$$

If the observer gain L makes $A_{a}-LC_{a}$ Hurwitz, then for any symmetric positive-definite matrix Q, there exists a symmetric positive-definite matrix P satisfying:

(44) $${\left(A_{a}-LC_{a}\right)}^{T}P+P(A_{a}-LC_{a})=-Q$$

The Lyapunov function is selected as:

(45) $$V=e^{T}Pe$$

Taking the derivative of V and substituting Equation (43) yields:

(46) $$\dot{V}=-e^{T}Qe+2e^{T}Pd_{o}$$

Using the norm inequality, one obtains:

(47) $$\dot{V}\leq -\lambda_{\min}(Q)\|e{\|}^{2}+2\|e\|\|P\|{\bar{d}}_{o}$$

Therefore, when the error norm satisfies:

(48) $$\|e\|>\frac{2\|P\|{\bar{d}}_{o}}{\lambda_{\min}(Q)}$$

there is $\dot{V}<0$. This leads to the following result.

**Theorem 1.**  *If the variation rate of the equivalent neighboring flow disturbance and the mechanical-side load deviation are bounded, the augmented system *$\left(A_{a},C_{a}\right)$ *is detectable, and the observer gain *L *makes *$A_{a}-LC_{a}$ *Hurwitz, then the error system of the extended state observer defined by Equation (35) is uniformly ultimately bounded. In particular, when *$d_{o}=0$, *the observation error asymptotically converges to zero.*

This result indicates that, under bounded neighboring supply–return disturbance variations and finite mechanical-side load disturbances, the designed observer can provide bounded estimates of the common boundaries and disturbance variables for the output-feedback controller.

Transient peaks in the observation error mainly occur during rapid changes in the neighboring-branch valve state. At these instants, the local linearized model cannot fully reproduce the square-root valve-flow nonlinearity, pipeline inertance effect, pressure-propagation delay, and measurement noise. These effects enter the error dynamics through the bounded term $d_{o}$ in Equation (42). Since the output-feedback MPC uses input-increment constraints and output soft constraints, the amplification of short-duration observation-error peaks into the valve-control input is limited. Their influence on the closed-loop response is further evaluated in the simulation and experimental results in Section 5.

### 3.5. Discrete Implementation and Control Interface

To interface with the discrete receding-horizon optimization of the model predictive controller in the next section, the continuous-time augmented system is discretized. Let the sampling period be $T_{s}$. The discrete augmented system is then written as:

(49) $$\xi (k+1)=A_{ad}\xi (k)+B_{ad}\Delta u(k)+G_{ad}\Delta F_{d}(k)+E_{ad}\nu (k)$$

The output equation is:

(50) $$\Delta Y(k)=C_{a}\xi (k)$$

where $A_{ad}$, $B_{ad}$, $G_{ad}$, and $E_{ad}$ are the augmented state matrix, input matrix, load-disturbance matrix, and neighboring-disturbance variation-rate matrix obtained by discretizing the continuous augmented system, respectively. $C_{a}$ is the output matrix of the augmented system.

The discrete extended state observer adopts a prediction–correction form:

(51) $${\hat{\xi }}^{-}(k+1)=A_{ad}\hat{\xi }(k)+B_{ad}\Delta u(k)$$

(52) $$\hat{\xi }(k+1)={\hat{\xi }}^{-}(k+1)+L_{d}\left[\Delta Y(k+1)-C_{a}{\hat{\xi }}^{-}(k+1)\right]$$

where $L_{d}$ is the discrete observer gain matrix. Through Equations (51) and (52), the augmented-state estimate can be obtained at each sampling instant, leading to:

(53) $$\hat{X}(k)=\bar{X}(k)+\Delta \hat{X}(k)$$

(54) $$\hat{\omega }(k)=\bar{\omega }(k)+\Delta \hat{\omega }(k)$$

The boundary states and disturbance reconstruction results actually used by the controller are:

(55) $${\hat{X}}_{c}(k)={\left[\begin{array}{cccc} {\hat{P}}_{s}(k) & {\hat{P}}_{r}(k) & {\hat{q}}_{n,s}(k) & {\hat{q}}_{n,r}(k) \end{array}\right]}^{T}$$

In Section 4, ${\hat{P}}_{s}$, ${\hat{P}}_{r}$, ${\hat{q}}_{n,s}$, and ${\hat{q}}_{n,r}$ are used to replace the common supply–return pressure boundaries and equivalent neighboring disturbances that are difficult to measure directly. These reconstructed variables are then introduced into the output-feedback prediction model. In this way, the control structure follows the sequence of target-cylinder-side measurement, common-boundary observation, receding-horizon prediction, and constrained optimization.

### 3.6. Interpretation of Equivalent Disturbance Estimates

In the augmented observer, ${\hat{q}}_{n,s}$ and ${\hat{q}}_{n,r}$ are reconstructed as equivalent inputs associated with the supply-node and return-node continuity equations. Their signs are consistent with the corresponding pressure-boundary variations. A positive $q_{n,s}$ represents a reduction in neighboring supply-side flow demand and tends to increase $P_{s}$, while a negative $q_{n,s}$ represents increased neighboring supply-side flow demand and tends to reduce $P_{s}$. A positive $q_{n,r}$ represents additional neighboring discharge entering the common return node and tends to increase $P_{r}$.

The reconstructed disturbance estimates are interpreted within the control-oriented model in Section 2. They summarize the effect of neighboring support actions on the local supply and return pressure dynamics and are used to update the prediction model in the output-feedback MPC. Their values depend on the adopted model structure, operating-point linearization, measurement outputs, and observer gain. This interpretation is consistent with the bounded-error result in Section 3.4 and with the controller-interface variable $X_{c}$ defined in Equation (23).

## 4. Observer-Based Output-Feedback Model Predictive Control Design

The extended state observer developed in Section 3 can estimate the system state $\hat{X}$, the neighboring disturbance $\hat{\omega }$, and the controller-interface variable ${\hat{X}}_{c}={\left[{\hat{P}}_{s},{\hat{P}}_{r},{\hat{q}}_{n,s},{\hat{q}}_{n,r}\right]}^{T}$ online by using the measurable target-cylinder outputs $Y={\left[x,v,P_{a},P_{b}\right]}^{T}$. Based on these estimates, this section develops an output-feedback model predictive controller. The reconstructed common supply–return boundaries and neighboring disturbances are embedded into the prediction model, and the constrained optimization problem is solved in a receding-horizon manner under input-amplitude, input-increment, and output soft constraints.

The proposed controller uses target-cylinder measurements and observer-reconstructed boundary variables to construct the prediction model. Direct measurements of the common supply-node pressure, common return-node pressure, and neighboring-branch flow rates are not required. State completion and disturbance compensation are achieved through the extended-state-observer–output-feedback-MPC structure, which supports controller implementation under limited measurement conditions.

### 4.1. Prediction Model Based on Observed States

As described in Section 3, the discrete deviation model of the system around the current operating point can be written as:

(56) $$\Delta X(k+1)=A_{d}\Delta X(k)+B_{d}\Delta u(k)+E_{d}\Delta \omega (k)+G_{d}\Delta F_{d}(k)$$

where ΔX(k) is the system-state deviation, Δu(k) is the valve-control input deviation, Δω(k) is the equivalent neighboring supply–return disturbance deviation, and $\Delta F_{d}(k)$ is the lumped mechanical-side load deviation. The matrices $A_{d}$, $B_{d}$, $E_{d}$, and $G_{d}$ are obtained by discretizing the local linearized model in Section 3.

Since $P_{s}$, $P_{r}$, $q_{n,s}$, and $q_{n,r}$ are difficult to measure directly, the observer outputs are used to construct the prediction model [23]. From Section 3, one obtains:

(57) $$\hat{X}(k)=\bar{X}(k)+\Delta \hat{X}(k),\;\hat{\omega }(k)=\bar{\omega }(k)+\Delta \hat{\omega }(k)$$

The estimated boundary states and disturbance variables called by the controller are:

(58) $${\hat{X}}_{c}(k)={\left[\begin{array}{cccc} {\hat{P}}_{s}(k) & {\hat{P}}_{r}(k) & {\hat{q}}_{n,s}(k) & {\hat{q}}_{n,r}(k) \end{array}\right]}^{T}$$

By replacing the ideal full-state deviation ΔX(k) with the observed state deviation $\Delta \hat{X}(k)$, and replacing the unmeasured disturbance deviation Δω(k) with the observed disturbance deviation $\Delta \hat{\omega }(k)$, the output-feedback prediction model is obtained as:

(59) $$\Delta \hat{X}(k+1)=A_{d}\Delta \hat{X}(k)+B_{d}\Delta u(k)+E_{d}\Delta \hat{\omega }(k)$$

In Equation (59), $\Delta F_{d}(k)$ is not explicitly introduced into the prediction model, but is treated as a bounded model disturbance in the subsequent closed-loop boundedness analysis. In this prediction model, the reconstructed common-node pressures and equivalent neighboring-disturbance flows provide the boundary and disturbance information required for receding-horizon prediction. Thus, the observer output is used to update the prediction dynamics at each sampling instant, and the effect of shared supply–return coupling is considered in the constrained optimization process.

To limit abrupt spool motion and improve input smoothness, the control increment is defined as:

(60) δu(k)=Δu(k)−Δu(k−1)

The augmented prediction state is further constructed as:

(61) $$\eta (k)={\left[\begin{array}{cc} \Delta {\hat{X}}^{T}(k) & \Delta u^{T}(k-1) \end{array}\right]}^{T}$$

Then Equation (59) can be rewritten as an augmented prediction model with δu(k) as the optimization variable:

(62) $$\eta (k+1)=\bar{A}\eta (k)+\bar{B}\delta u(k)+\bar{E}\Delta \hat{\omega }(k)$$

where:

(63) $$\bar{A}=\left[\begin{array}{cc} A_{d} & B_{d}\\ 0 & I \end{array}\right],\;\bar{B}=\left[\begin{array}{c} B_{d}\\ I \end{array}\right],\;\bar{E}=\left[\begin{array}{c} E_{d}\\ 0 \end{array}\right]$$

The control objective includes displacement tracking, supply-pressure fluctuation suppression, and return-backpressure fluctuation suppression. Therefore, the performance output is defined as:

(64) $$Z(k)=C_{z}\eta (k)$$

where:

(65) $$Z(k)={\left[\begin{array}{ccc} \Delta x(k) & \Delta P_{s}(k) & \Delta P_{r}(k) \end{array}\right]}^{T}$$

The output-selection matrix is:

(66) $$C_{z}=\left[\begin{array}{ccccccccc} 1 & 0 & 0 & 0 & 0 & 0 & 0 & 0 & 0\\ 0 & 0 & 0 & 0 & 1 & 0 & 0 & 0 & 0\\ 0 & 0 & 0 & 0 & 0 & 0 & 1 & 0 & 0 \end{array}\right]$$

Equations (64)–(66) indicate that the controller simultaneously considers the target displacement deviation, common supply-node pressure deviation, and common return-node pressure deviation during prediction. This avoids a control action that only pursues displacement tracking while neglecting fluctuations in the supply–return pressure boundaries.

Let the prediction horizon be $N_{p}$ and the control horizon be $N_{c}$. The control-increment sequence is defined as:

(67) $$\Delta U(k)={\left[\begin{array}{cccc} \delta u(k) & \delta u(k+1) & \cdots & \delta u(k+N_{c}-1) \end{array}\right]}^{T}$$

The neighboring-disturbance prediction sequence is defined as:

(68) $$\Delta \Omega (k)={\left[\begin{array}{cccc} \Delta {\hat{\omega }}^{T}(k) & \Delta {\hat{\omega }}^{T}(k+1) & \cdots & \Delta {\hat{\omega }}^{T}(k+N_{p}-1) \end{array}\right]}^{T}$$

If the neighboring action sequence or valve-control command is available, $\Delta \hat{\omega }$ can be extrapolated by combining the observer estimate with the action timing. If only the current estimate is available, a constant-hold approximation is used over the prediction horizon. By recursively applying Equation (62), the predicted performance-output sequence can be obtained as:

(69) $$Z_{p}(k)=\Phi \eta (k)+\Theta \Delta U(k)+\Gamma \Delta \Omega (k)$$

where $Z_{p}(k)$ is the performance-output sequence over the prediction horizon. Φ, Θ, and Γ are the prediction matrices constructed from A, B, E, and $C_{z}$.

### 4.2. Objective Function and Constraint Design

The control objectives of the output-feedback MPC include displacement tracking, supply-pressure fluctuation suppression, return-backpressure fluctuation suppression, and valve-input smoothing. The reference output sequence is defined as:

(70) $$Z_{r}(k)={\left[\begin{array}{cccc} Z_{r}^{T}(k+1) & Z_{r}^{T}(k+2) & \cdots & Z_{r}^{T}(k+N_{p}) \end{array}\right]}^{T}$$

where $Z_{r}$ includes the displacement reference deviation, supply-pressure reference deviation, and return-pressure reference deviation. In general, the reference deviations of the supply pressure and return pressure are set to zero, so that $P_{s}$ and $P_{r}$ are maintained near their nominal operating points.

The finite-horizon performance index is defined as [24]:

(71) $$J={\left[Z_{p}(k)-Z_{r}(k)\right]}^{T}Q\left[Z_{p}(k)-Z_{r}(k)\right]+\Delta U^{T}(k)R\Delta U(k)+\rho \varepsilon^{T}\varepsilon$$

where Q is the output-error weighting matrix, R is the input-increment weighting matrix, ε is the slack variable for output soft constraints, and ρ is the slack-variable penalty coefficient. The weighting matrices can be selected as:

(72) $$Q=\mathrm{diag}\left(Q_{1},Q_{2},\cdots ,Q_{N_{p}}\right)$$

(73) $$R=\mathrm{diag}\left(R_{1},R_{2},\cdots ,R_{N_{c}}\right)$$

where $Q_{i}$ coordinates the displacement deviation, supply-pressure deviation, and return-pressure deviation, while $R_{i}$ limits the variation rate of the control input.

Considering the amplitude limitation of the proportional-valve input, the following constraint is imposed:

(74) $$u_{\min}\leq u(k+i)\leq u_{\max}$$

Considering the spool-motion speed limitation, the input-increment constraint is imposed as:

(75) $$\delta u_{\min}\leq \delta u(k+i)\leq \delta u_{\max}$$

Over the prediction horizon, the input-deviation sequence can be obtained by accumulating the control-increment sequence:

(76) $$\Delta U_{a}(k)=S\Delta U(k)+1\Delta u(k-1)$$

where S is a lower triangular accumulation matrix:

(77) $$S=\left[\begin{array}{cccc} 1 & 0 & \cdots & 0\\ 1 & 1 & \cdots & 0\\ \vdots & \vdots & \ddots & \vdots \\ 1 & 1 & \cdots & 1 \end{array}\right]$$

Therefore, the input-amplitude constraints can be uniformly written as:

(78) $$U_{\min}\leq \Delta U_{a}(k)+\bar{U}\leq U_{\max}$$

The input-increment constraints can be written as:

(79) $$\Delta U_{\min}\leq \Delta U(k)\leq \Delta U_{\max}$$

For output constraints, bounds are imposed on the displacement deviation, supply-pressure deviation, and return-pressure deviation to ensure safe and controllable pushing:

(80) $$Z_{\min}-\varepsilon \leq Z_{p}(k)\leq Z_{\max}+\varepsilon$$

The slack variable is required to satisfy:

(81) ε≥0

Equation (80) incorporates the displacement deviation, supply-pressure fluctuation, and return-backpressure fluctuation into a unified constraint framework. The slack variable prevents short-term disturbances or model mismatch from making the optimization problem infeasible.

### 4.3. Quadratic Programming Formulation and Receding-Horizon Solution

Substituting Equation (69) into the performance index in Equation (71), and introducing the optimization variable:

(82) $$\Lambda ={\left[\begin{array}{cc} \Delta U^{T}(k) & \varepsilon^{T} \end{array}\right]}^{T}$$

the optimization problem at the current sampling instant can be written in the standard quadratic programming form:

(83) $$\min_{\Lambda }\;\frac{1}{2}\Lambda^{T}H\Lambda +g^{T}\Lambda$$

The constraints are uniformly expressed as:

(84) MΛ≤b

where the Hessian matrix is:

(85) $$H=2\left[\begin{array}{cc} \Theta^{T}Q\Theta +R & 0\\ 0 & \rho I \end{array}\right]$$

and the linear term is:

(86) $$g=2\left[\begin{array}{c} \Theta^{T}Q\left[\Phi \eta (k)+\Gamma \Delta \Omega (k)-Z_{r}(k)\right]\\ 0 \end{array}\right]$$

Equation (84) consists of the input-amplitude constraints, input-increment constraints, output soft constraints, and non-negativity constraints on the slack variables. Thus, the finite-horizon optimization problem is transformed into a quadratic programming problem with linear inequality constraints.

**Theorem 2.**  *If* Q≻0 *,* R≻0 *, and* ρ>0 *, then the Hessian matrix* H *in Equation (85) is symmetric positive definite. Therefore, when the feasible set is non-empty, the quadratic programming problem in Equation (83) is strictly convex and has a unique optimal solution.*

**Proof.**  Since Q≻0, it follows that $\Theta^{T}Q\Theta ≽0$. Because R≻0, one obtains $\Theta^{T}Q\Theta +R≻0$. Meanwhile, ρ>0 guarantees ρI≻0. Therefore, H≻0, and the optimization problem is a strictly convex quadratic program. This completes the proof. □

Let the optimal solution of the optimization problem be:

(87) $$\Lambda^{*}={\left[\begin{array}{cc} \Delta U^{*T}(k) & \varepsilon^{*T} \end{array}\right]}^{T}$$

where the optimal control-increment sequence is:

(88) $$\Delta U^{*}(k)={\left[\begin{array}{cccc} \delta u^{*}(k) & \delta u^{*}(k+1) & \cdots & \delta u^{*}(k+N_{c}-1) \end{array}\right]}^{T}$$

Following the receding-horizon principle of MPC, only the first optimal control increment is applied to the actual system:

(89) $$\Delta u(k)=\Delta u(k-1)+\delta u^{*}(k)$$

The actual valve-control input is then obtained as:

(90) $$u(k)=\bar{u}(k)+\Delta u(k)$$

At the next sampling instant, the observer updates $\hat{X}(k+1)$ and $\hat{\omega }(k+1)$ using the newly measured outputs. The controller then reconstructs the prediction model and solves a new quadratic programming problem, thereby forming a closed-loop receding-horizon optimization process.

### 4.4. Recursive Feasibility and Closed-Loop Boundedness Analysis

To characterize the basic closed-loop properties of the proposed output-feedback MPC, this subsection analyzes recursive feasibility and closed-loop boundedness [25]. The following analysis is carried out for the local linearized prediction model within the admissible operating region defined in Section 2.5. Because the hydraulic plant contains valve-flow nonlinearities, input saturation, pressure constraints, observation errors, and bounded unmodeled dynamics, global asymptotic stability of the complete nonlinear system is not claimed. The analysis focuses on local recursive feasibility and uniform ultimate boundedness under bounded observation errors and bounded disturbance variations.

If the optimization problem is feasible at sampling instant k, and the optimal control-increment sequence is given by Equation (88), then a candidate control sequence at the next sampling instant can be constructed as:

(91) $$\Delta \tilde{U}(k+1)={\left[\begin{array}{cccc} \delta u^{*}(k+1) & \cdots & \delta u^{*}(k+N_{c}-1) & 0 \end{array}\right]}^{T}$$

This candidate sequence is obtained by shifting the previous optimal solution by one step and appending zero at the terminal point. Since both the input-amplitude constraints and input-increment constraints are convex, and since non-negative slack variables are introduced into the output constraints, the shifted sequence provides a feasible candidate in a local neighborhood of the nominal operating trajectory under bounded observation errors, bounded disturbance-prediction errors, and bounded load disturbances. The output soft constraints are used to prevent temporary pressure-boundary deviations from causing infeasibility.

**Theorem 3.**  *If the optimization problem is feasible at the initial sampling instant, the extended state observer error is uniformly ultimately bounded, and the neighboring supply–return disturbance variations and mechanical-side load disturbances are bounded within a single prediction horizon, then the output-feedback MPC optimization problem with soft constraints is locally recursively feasible during closed-loop operation.*

Next, considering the effects of observation error, disturbance-prediction error, and load disturbance on the closed-loop system, the actual closed-loop augmented-state model can be written as:

(92) $$\eta (k+1)=\bar{A}\eta (k)+\bar{B}\delta u(k)+\bar{E}\Delta \hat{\omega }(k)+d_{c}(k)$$

where $d_{c}(k)$ denotes the bounded disturbance composed of observation error, disturbance-prediction error, model-linearization error, and mechanical-side load disturbance. It satisfies:

(93) $$\|d_{c}(k)\|\leq {\bar{d}}_{c}$$

Let the optimal value function at the current sampling instant be:

(94) $$V_{m}(k)=J^{*}(k)$$

Using the positive definiteness of the quadratic performance index and the shifted candidate sequence construction in MPC, one obtains:

(95) $$V_{m}(k+1)-V_{m}(k)\leq -\alpha_{1}\|Z(k)-Z_{r}(k){\|}^{2}-\alpha_{2}\|\delta u(k){\|}^{2}+\alpha_{3}{\bar{d}}_{c}^{2}$$

where $\alpha_{1}$, $\alpha_{2}$, and $\alpha_{3}$ are positive constants related to the system matrices and weighting matrices. Equation (95) shows that the value function decreases along the closed-loop trajectory when the output error and control increment are sufficiently large. In the presence of bounded disturbances, the closed-loop state ultimately converges to a bounded neighborhood of the origin.

**Theorem 4.**  *Under the conditions of Theorems 2 and 3, if the extended state observer error is uniformly ultimately bounded and the closed-loop disturbance *$d_{c}(k)$ *is bounded, then the state, output, and control input of the closed-loop system composed of the extended state observer and output-feedback MPC are uniformly ultimately bounded.*

In summary, the proposed LESO-OFMPC first updates $\hat{X}(k)$, $\hat{\omega }(k)$, and ${\hat{X}}_{c}(k)$ at each sampling instant using the measurable output Y(k). It then constructs the prediction model based on the observed states and disturbance estimates, solves the constrained quadratic programming problem, and applies only the first optimal control increment to the proportional valve of the target branch. This receding-horizon mechanism couples the observer and predictive controller under limited measurement conditions, enabling common supply–return boundary reconstruction, neighboring-disturbance compensation, and constrained optimization of the valve-control input.

The online computational burden of the proposed controller is mainly determined by the solution of the quadratic programming problem in Equation (83). For fixed prediction and control horizons, the dimensions of the Hessian matrix and inequality constraints remain constant during closed-loop operation. Therefore, the computational delay can be evaluated by recording the average and maximum QP solution times at each sampling instant. The computational burden and implementation-related issues are discussed in Section 5.

## 5. Co-Simulation and Hydraulic-Platform Experimental Validation

To validate the proposed supply–return disturbance coupling model, extended state observer, and output-feedback model predictive control method, an AMESim–MATLAB/Simulink co-simulation platform and a dual-branch hydraulic experimental platform are developed. AMESim is used to model the dual-branch hydraulic system with shared supply and return circuits, while MATLAB/Simulink implements the extended state observer, prediction-model update, and receding-horizon optimization of the output-feedback MPC. The validation consists of two parts. The first examines the reconstruction performance of the extended state observer for the common supply-node pressure $P_{s}$, common return-node pressure $P_{r}$, and equivalent neighboring disturbances $q_{n,s}$ and $q_{n,r}$. The second compares the velocity stability, pressure disturbance response, and input smoothness of different control methods under coupled supply–return disturbances induced by rapid valve closure in the disturbance branch.

Unlike the equivalent disturbance modeling in Section 2, the co-simulation does not artificially impose $q_{n,s}$ and $q_{n,r}$ as separate external inputs. Instead, the coupled disturbance is generated naturally by the valve opening and closing of the disturbance branch and by the dynamics of the shared supply–return pipelines. After rapid valve closure in the disturbance branch, both the supply-flow demand and the return-flow state change simultaneously, leading to transient fluctuations in $P_{s}$ and $P_{r}$. Therefore, this operating condition reflects the practical coupling effect of neighboring actuator actions on the target branch in a multi-actuator hydraulic system with shared supply and return circuits.

### 5.1. Co-Simulation Platform and Disturbance Condition

**Figure 1:**
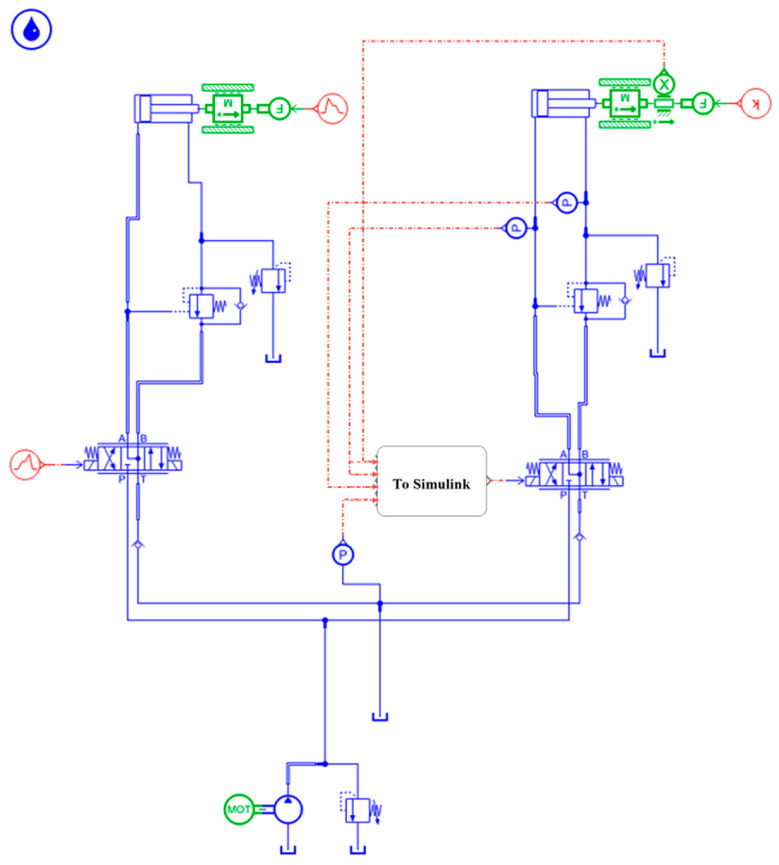
AMESim–MATLAB/Simulink co-simulation model.

Figure 1 shows the AMESim–MATLAB/Simulink co-simulation model [26]. The hydraulic plant is established in AMESim and mainly includes a constant-pressure pump source, a common supply main, a common return main, the proportional valve of the target branch, the target hydraulic cylinder, the proportional valve of the disturbance branch, the disturbance hydraulic cylinder, and the mechanical load. The target branch represents the controlled pushing cylinder, while the disturbance branch represents start–stop or valve-closure actions of a neighboring actuator. The two branches share the same supply main and common return main; therefore, the disturbance-branch action can affect the dynamic response of the target branch through the common pressure boundaries [27,28]. The control algorithms are implemented in MATLAB/Simulink, including the extended state observer, the output-feedback MPC solver, and the PID, MPC, and DOB-MPC controllers used for comparison.

During simulation, the total duration is set to T=10 s. The disturbance branch begins loading at t=0 s to establish the operating state of a neighboring actuator in the shared supply–return system. The target branch starts the pushing-load task at t=3 s. At t=5 s, the disturbance branch closes rapidly, causing a sudden change in the supply-side flow demand and the return-side flow state. This produces coupled supply–return disturbances acting on the target branch. After the disturbance occurs, the target branch continues operating until the end of the simulation, so that the post-disturbance recovery and stability of the controller can be evaluated.

For fair comparison, the PID, MPC, DOB-MPC, and LESO-OFMPC controllers were tested under the same disturbance timing and steady-state operating condition. The target branch started at t=3.0 s, and the disturbance branch closed rapidly at t=5.0 s. The fixed-step solver step and response recording interval were both set to 0.001 s, while the control update period of the predictive controllers was set to $T_{s}=0.01\;s$. The valve input was constrained within u∈[0,40], and the input increment was limited by Δu∈[−0.8,0.8] at each control update. The PID gains were selected as $K_{p}=420$, $K_{i}=85$, and $K_{d}=1.2$, with the derivative filter coefficient set to N=80. For the MPC-based controllers, the prediction horizon and control horizon were set to $N_{p}=40$ and $N_{c}=10$, respectively. The output-weighting matrix was selected as $Q_{y}=\mathrm{diag}(12,3,3)$ for the normalized output vector ${\left[x,P_{s},P_{r}\right]}^{T}$, the input-increment weight was set to $R_{\Delta u}=0.35$, and the slack-variable penalty was set to $\rho =1.0\times 10^{5}$. For DOB-MPC, a first-order disturbance-observer filter $Q_{d}(s)=1/(\tau_{d}s+1)$ with $\tau_{d}=0.025\;s$ was used to estimate the lumped disturbance term. For the proposed LESO-OFMPC, the LESO bandwidth was set to $\omega_{o}=85\;\mathrm{rad}/s$, and the reconstructed controller-interface vector was ${\hat{X}}_{c}={\left[{\hat{P}}_{s},{\hat{P}}_{r},{\hat{q}}_{n,s},{\hat{q}}_{n},r\right]}^{T}$.

### 5.2. Reconstruction Performance of Common Supply–Return Boundaries and Neighboring Disturbances

To verify the reconstruction capability of the extended state observer, the true variables obtained from the co-simulation model are used as references and compared with the observer estimates. The observer inputs are the displacement, velocity, and two chamber pressures of the target hydraulic cylinder. The variables to be reconstructed are the common supply-node pressure $P_{s}$, the common return-node pressure $P_{r}$, and the equivalent neighboring supply–return disturbances $q_{n,s}$ and $q_{n,r}$. The root mean square error, mean absolute error, maximum absolute error, and convergence time within the 5% error band are adopted as quantitative indices. The RMSE is sensitive to peak errors during disturbance transients, whereas the MAE reflects the average deviation of the estimate from the true value. The reference values of $P_{s}$, $P_{r}$, $q_{n,s}$, and $q_{n,r}$ are extracted from the co-simulation model and are used only for offline evaluation of the observer reconstruction performance.

**Figure 2:**
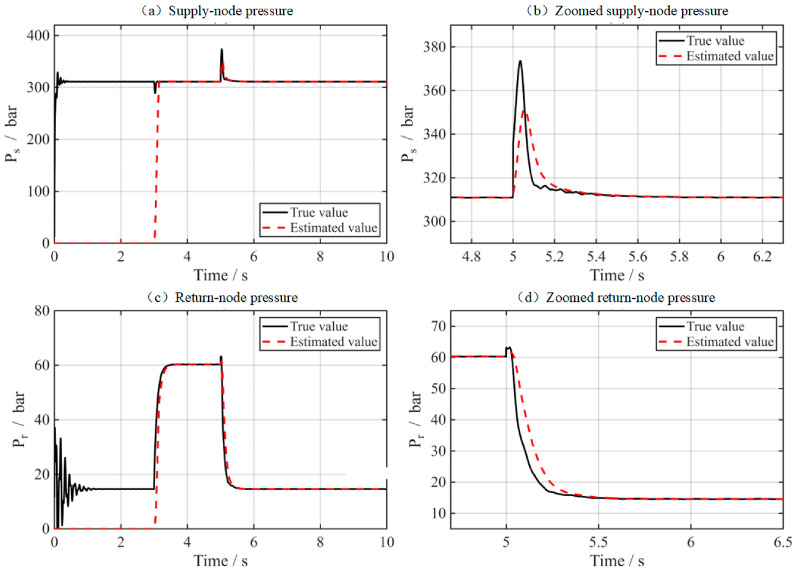
Comparison of estimated common supply–return-node pressures.

Figure 2 compares the true and estimated values of $P_{s}$ and $P_{r}$. Figure 2a,c show the full-time responses of $P_{s}$ and $P_{r}$, respectively, while Figure 2b,d provide local enlarged views during valve closure in the disturbance branch. The estimates exhibit short transient deviations during system start-up and initial target-branch loading, but then converge rapidly. When the disturbance branch closes rapidly at t=5 s, the reduced supply-side flow demand causes a transient rise in $P_{s}$; meanwhile, the sudden change in the return-flow state leads to a rapid decrease and subsequent recovery of $P_{r}$. In the interval t=5.0–6.5 s, the RMSE and MAE of the $P_{s}$ estimation error are 6.1776 bar and 1.9151 bar, respectively. The maximum absolute error is 34.7193 bar, and the convergence time into the 5% error band is approximately 0.156 s. For $P_{r}$, the RMSE and MAE are 3.6718 bar and 1.4518 bar, respectively; the maximum absolute error is 15.4896 bar, and the convergence time into the 5% error band is approximately 0.257 s. These results indicate that the proposed observer can capture the dominant dynamic variations in the common supply–return pressure boundaries within a short period after disturbance transients.

**Figure 3:**
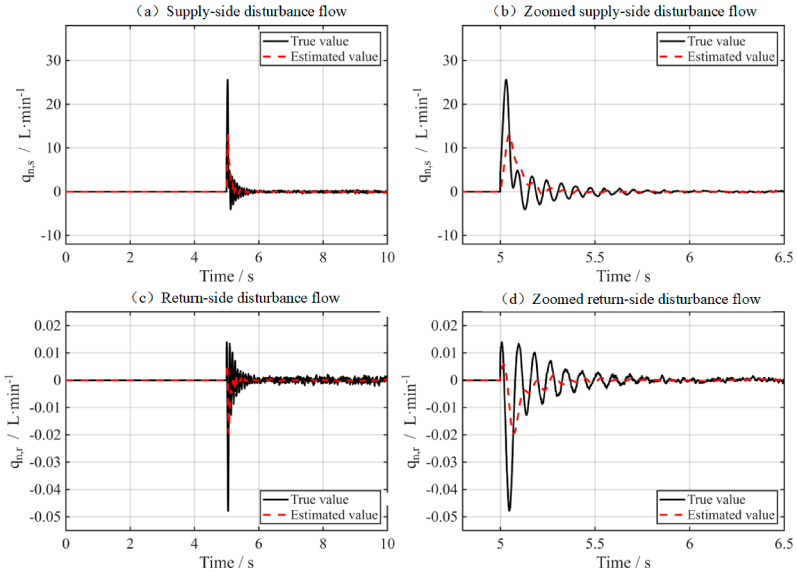
Comparison of estimated neighboring supply–return disturbances.

Figure 3 compares the true and estimated values of $q_{n,s}$ and $q_{n,r}$. Figure 3a,c show the full-time responses of $q_{n,s}$ and $q_{n,r}$, respectively, while Figure 3b,d provide local enlarged views during the valve-closure stage of the disturbance branch. It should be noted that $q_{n,s}$ and $q_{n,r}$ are not the original flow rates in the common supply and return mains, but equivalent disturbance components generated by the disturbance-branch action relative to the nominal flow. Therefore, both variables mainly exhibit transient variations near t=5 s and gradually decay after the system re-establishes flow balance. In the interval t=5.0–6.5 s, the RMSE and MAE of the $q_{n,s}$ estimation error are 2.6362 L/min and 1.0644 L/min, respectively. The maximum absolute error is 16.3958 L/min, and the convergence time into the 5% error band is approximately 0.438 s. For $q_{n,r}$, the RMSE and MAE are 0.0066 L/min and 0.0028 L/min, respectively; the maximum absolute error is 0.0400 L/min, and the convergence time into the 5% error band is approximately 0.566 s. These results show that the observer exhibits a degree of underestimation and delay at the disturbance peak, but it accurately captures the disturbance direction, active interval, and decay trend.

The pressure-boundary estimation errors are mainly concentrated near the target-branch start instant and the disturbance-branch valve-closure instant. At these instants, the valve-orifice flow changes rapidly, and the local linearized observer model cannot fully reproduce the square-root flow nonlinearity, pipeline inertance effect, pressure-propagation delay, and measurement noise. Therefore, the maximum errors of $P_{s}$ and $P_{r}$ appear as short-duration transient peaks. After the transient interval, the estimated pressure boundaries follow the reference curves, indicating that the observer captures the dominant variations in the common supply and return pressure boundaries required by the prediction model.

Overall, the extended state observer can reconstruct the common supply–return pressure boundaries and equivalent neighboring disturbances online using only measurable information from the target hydraulic cylinder. It therefore provides effective boundary-state and disturbance-compensation information for the subsequent output-feedback MPC.

The reconstructed equivalent disturbance flows show clear transient responses around the disturbance-branch valve-closure instant. The peak of ${\hat{q}}_{n,s}$ occurs together with the rapid variation in the supply-node pressure, while the variation of ${\hat{q}}_{n,r}$ is synchronized with the return-node pressure transient. After the post-closure transition, both estimated disturbance flows converge toward zero, which is consistent with their definition as equivalent nodal disturbance inputs relative to the post-closure operating condition. These results support the interpretation that $q_{n,s}$ and $q_{n,r}$ represent the dominant influence of neighboring support actions on the local supply and return pressure dynamics within the control-oriented model.

### 5.3. Comparison of Control Strategies and Supply–Return Disturbance Suppression

The comparative evaluation includes PID, conventional MPC, disturbance-observer-based MPC (DOB-MPC), and the proposed LESO-OFMPC under the same disturbance timing and operating condition. The DOB-MPC uses the same prediction horizon, control horizon, weighting matrices, and input constraints as the conventional MPC and the proposed LESO-OFMPC. A first-order disturbance observer is used to estimate a lumped disturbance term, which is then incorporated into the prediction model. The comparison evaluates the effect of structured supply–return disturbance reconstruction relative to lumped disturbance compensation.

**Figure 4:**
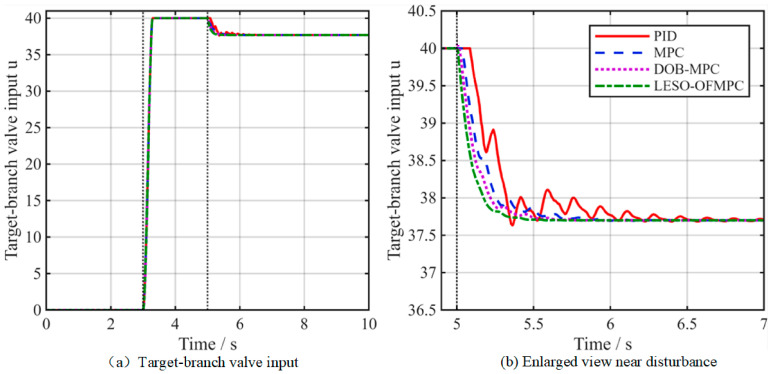
Comparison of target-branch valve-control inputs under different control methods.

Figure 4 compares the target-branch valve-control inputs under the four control methods. When the disturbance branch closes rapidly at t=5.0 s, an abrupt disturbance is imposed on the shared supply–return boundaries. The PID controller mainly relies on error feedback, resulting in a relatively large adjustment and residual fluctuation of the target-valve input after the disturbance. The conventional MPC improves input smoothness through the input-increment constraint, but it still lacks direct information on the common pressure boundaries and neighboring-branch disturbance. The DOB-MPC further reduces the input fluctuation by compensating for a lumped disturbance term. The proposed LESO-OFMPC gives the smallest input total variation because the observer reconstructs ${\hat{P}}_{s}$, ${\hat{P}}_{r}$, ${\hat{q}}_{n,s}$, and ${\hat{q}}_{n,r}$ and supplies these variables to the prediction model.

**Figure 5:**
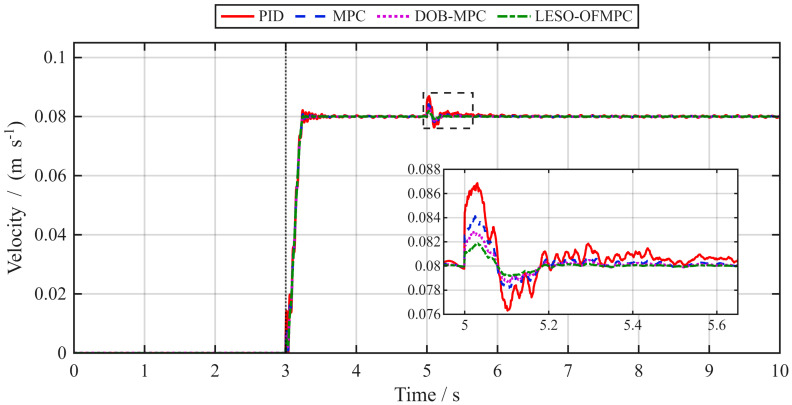
Comparison of velocity responses under different control methods.

Figure 5 shows the velocity response of the target hydraulic cylinder under different control methods. After valve closure in the disturbance branch, the supply-side flow demand decreases, causing a transient rise in the supply-node pressure. At the same time, the variation in the common return pressure changes the discharge pressure difference in the rod-end chamber, leading to transient velocity fluctuation of the target hydraulic cylinder. The PID controller produces the largest velocity deviation because it cannot predict the supply–return boundary variation. The conventional MPC suppresses part of the velocity fluctuation by constrained receding-horizon optimization. The DOB-MPC further reduces the post-disturbance velocity deviation through lumped disturbance compensation. The proposed LESO-OFMPC gives the smallest velocity deviation and the shortest recovery time because the reconstructed common-node pressures and equivalent neighboring-disturbance flows are introduced into the prediction model.

**Figure 6:**
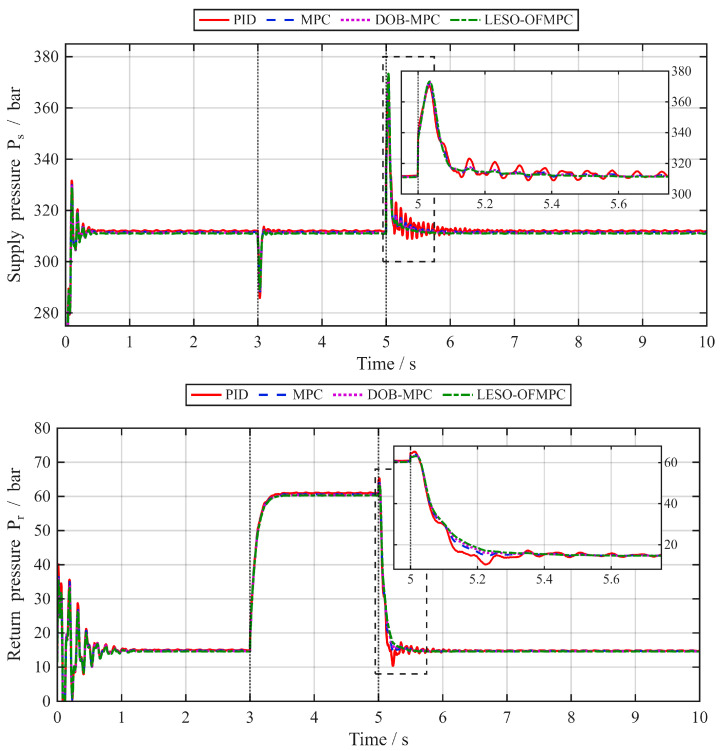
Comparison of supply–return pressure responses under different control methods.

Figure 6 compares the responses of $P_{s}$ and $P_{r}$ under the four control methods. After rapid valve closure in the disturbance branch, $P_{s}$ exhibits a transient overshoot, while $P_{r}$ undergoes a rapid transition and residual fluctuation. The peak of the common supply pressure is mainly associated with the disturbance-branch valve closure, pipeline inertance, and fluid compressibility, and the target-branch controller has limited authority over this instantaneous peak. Therefore, the difference among the controllers is mainly reflected in the post-peak recovery process, return-pressure fluctuation, velocity continuity, and valve-input smoothness. Compared with PID and conventional MPC, DOB-MPC reduces the residual pressure fluctuation by introducing lumped disturbance compensation. The proposed LESO-OFMPC further improves the coordinated response because the supply-side and return-side boundary variables are reconstructed and used in the prediction model.

Table 1 lists the disturbance-suppression performance indices of the four control methods. The maximum velocity deviations of PID, MPC, DOB-MPC, and LESO-OFMPC are 6.8403 mm/s, 4.1195 mm/s, 2.8058 mm/s, and 1.7484 mm/s, respectively, corresponding to velocity deviation percentages of 8.5479%, 5.1487%, 3.5073%, and 2.1854%. The recovery time of LESO-OFMPC within the 1% velocity error band is 0.112 s, which is shorter than 0.563 s for PID, 0.167 s for MPC, and 0.161 s for DOB-MPC. The input total variation in LESO-OFMPC is 2.3383, which is lower than 5.9635 for PID, 2.8808 for MPC, and 2.5585 for DOB-MPC. These results show that DOB-MPC improves the disturbance response compared with conventional MPC, while LESO-OFMPC gives the best overall performance in velocity-continuity preservation and valve-input smoothing under shared supply–return disturbances.

### 5.4. Hydraulic Experimental Platform Validation

**Figure 7:**
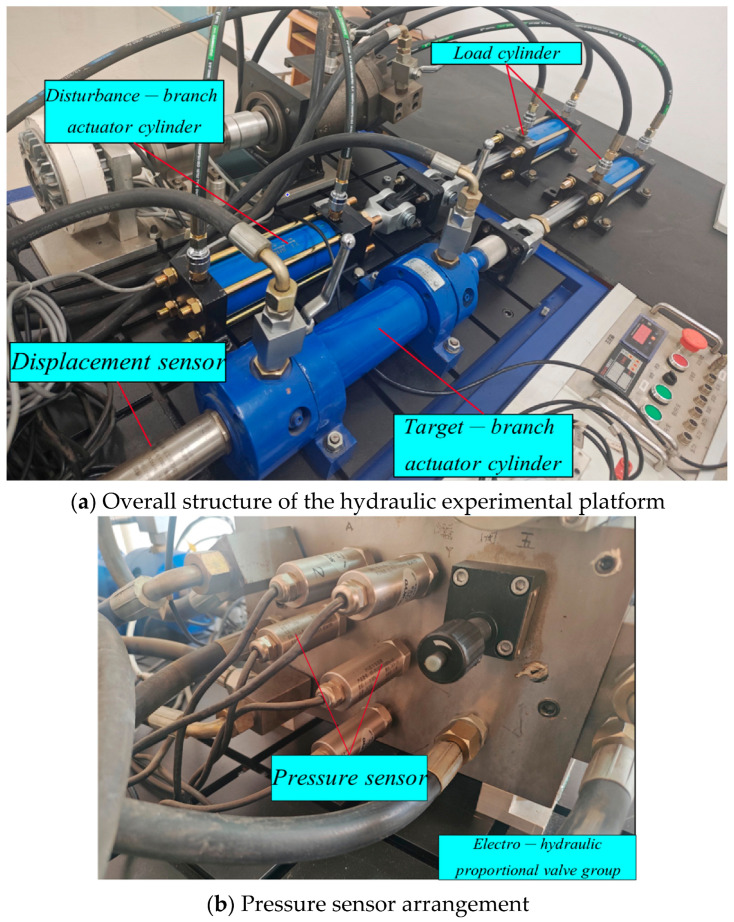
Dual-branch hydraulic experimental platform with common hydraulic-circuit coupling and sensor arrangement.

A dual-branch hydraulic experimental platform with common hydraulic-circuit coupling is used to evaluate the influence of coupled supply–return disturbances on the motion response of the target branch and the engineering applicability of the proposed method, as shown in Figure 7. The experimental platform consists of a hydraulic power source, electro-hydraulic proportional-valve groups, the working cylinder of the target circuit, the working cylinder of the disturbance circuit, load cylinders, displacement sensors, pressure sensors, and a data-acquisition and control system. The target circuit represents the controlled pushing actuator, while the disturbance circuit represents the action of a neighboring support. The two circuits share the same hydraulic source and common return channel.

Two operating conditions are considered: the fixed-opening condition and the controller-intervention condition. Under the fixed-opening condition, the valve-control input of the target circuit is held constant, and the target cylinder reaches a stable pushing velocity of approximately 10 mm/s before the disturbance. Under the controller-intervention condition, the target valve-control input is calculated online by the proposed output-feedback predictive controller. In both conditions, the disturbance circuit closes rapidly near t=5 s, ensuring consistent external disturbance conditions.

**Figure 8:**
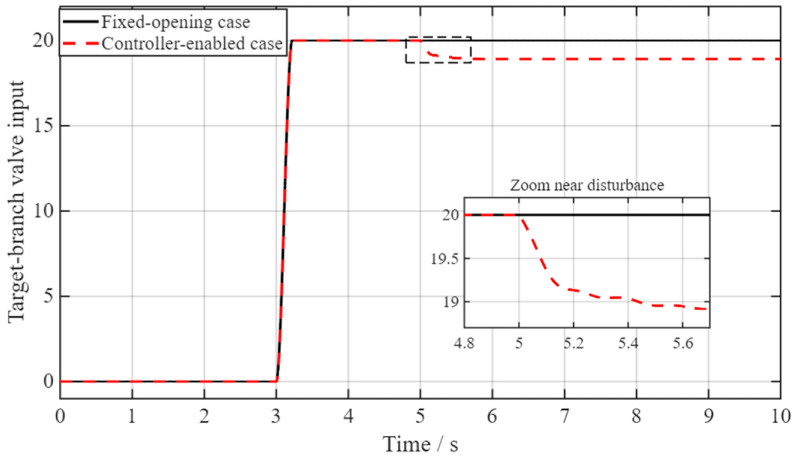
Comparison of valve-control inputs under the fixed-opening and controller-intervention conditions.

Figure 8 shows the target-valve input under the two conditions. Under the fixed-opening condition, the target-valve input remains unchanged during the pushing process and cannot actively adjust the valve opening in response to variations in the supply–return boundaries. Under the controller-intervention condition, the target-valve input is actively corrected after the disturbance occurs, indicating that the controller can adjust the valve-control input online according to the motion state and pressure feedback.

**Figure 9:**
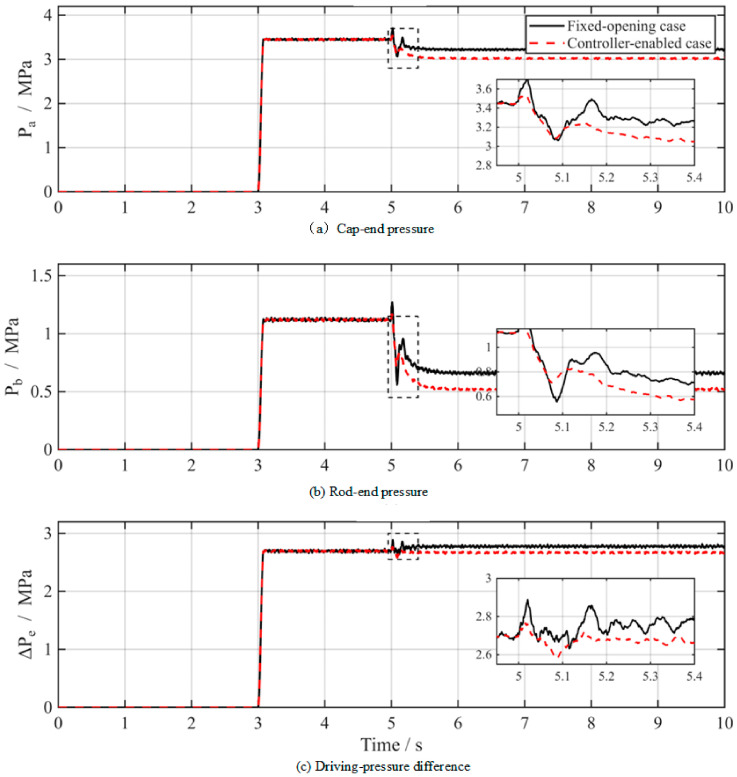
Comparison of two chamber pressures and equivalent driving-pressure difference in the target cylinder under the fixed-opening and controller-intervention conditions.

Figure 9 shows the two chamber pressures and equivalent driving-pressure difference in the target cylinder. Since the experimental platform does not use independent high-frequency pressure sensors at the common supply node and common return node, $P_{s}$ and $P_{r}$ are not directly measured in the experiment. Instead, the cap-end pressure $P_{a}$, rod-end pressure $P_{b}$, and equivalent driving-pressure difference $\Delta P_{e}$ are used to characterize the pressure response after supply–return disturbances are transmitted to the target actuator. After rapid valve closure in the disturbance circuit, $P_{a}$, $P_{b}$, and $\Delta P_{e}$ all exhibit transient fluctuations under the fixed-opening condition. After controller intervention, the target-valve input is corrected according to the feedback state, reducing the fluctuation of the equivalent driving-pressure difference and producing a smoother pressure recovery process. Therefore, the experimental pressure results are used to evaluate the branch-level pressure response after shared-circuit disturbance transmission, while the direct reconstruction accuracy of $P_{s}$ and $P_{r}$ is evaluated in the co-simulation results in Section 5.2.

**Figure 10:**
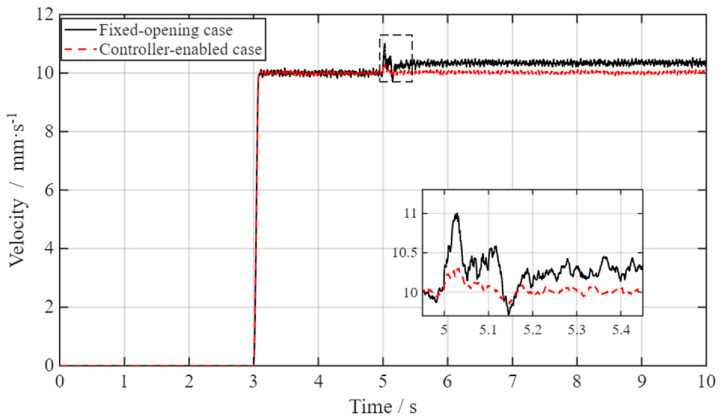
Comparison of target-cylinder velocity responses under the fixed-opening and controller-intervention conditions.

Figure 10 shows the target-cylinder velocity response under the two conditions. Under the fixed-opening condition, the target-cylinder velocity exhibits a distinct transient fluctuation after the disturbance, indicating that the target circuit cannot effectively resist common-circuit boundary disturbances without active regulation. After controller intervention, the target-valve input adjusts with the disturbance variation, substantially reducing the velocity fluctuation amplitude and shortening the recovery time.

Table 2 compares the experimental indices under the two conditions. After controller intervention, the maximum velocity fluctuation decreases from 1.0041 mm/s to 0.3011 mm/s, the velocity fluctuation percentage decreases from 10.0434% to 3.0111%, and the velocity recovery time decreases from 0.505 s to 0.038 s. The peak deviation of the equivalent driving-pressure difference is also reduced from 0.1933 MPa to 0.1107 MPa. These results demonstrate that the proposed method can mitigate the impact of valve-closure disturbances from the disturbance circuit on the motion continuity and driving-pressure stability of the target circuit in the actual hydraulic platform.

The dual-branch platform represents a branch-level coupling unit under a neighboring valve-closure disturbance. The validation focuses on the local interaction between one target branch and one disturbance branch through the shared hydraulic circuit. In a full support-group system, the local coupling unit extends to multiple neighboring supports, action scheduling, communication delay, and distributed controller deployment.

### 5.5. Robustness and Implementation Analysis

On the basis of the co-simulation and hydraulic-platform validation described above, the robustness and implementation characteristics of the proposed LESO-OFMPC are further analyzed. The closed-loop LESO-OFMPC validation model uses the same controller parameters as those in Section 5.3 and retains the same neighboring valve-closure disturbance condition. Parameter perturbation, measurement noise, measurement delay, and finite-horizon QP computation are examined to evaluate the influence of model-parameter variation, sensor uncertainty, and implementation-related factors on the closed-loop response.

The tested cases are summarized in Table 3. In the nominal case, the maximum velocity deviation after the neighboring valve-closure disturbance is 1.875 mm/s, with a velocity RMSE of 0.291 mm/s and a recovery time of 0.076 s. For parameter sensitivity, the hydraulic response time constant, valve-flow gain, and equivalent supply–return disturbance gain were perturbed by ±20% to evaluate the influence of aggregate hydraulic-response and valve-flow variations on the closed-loop performance. The worst tested condition is the combined adverse case, where the hydraulic time constant is decreased by 20%, the disturbance gain is increased by 20%, and the valve-flow gain is increased by 20%. Under this case, the maximum velocity deviation increases to 2.344 mm/s, the velocity RMSE is 0.343 mm/s, and the controller recovers to the 1% velocity band within 0.080 s. No valve-input constraint violation is observed in these tests.

For measurement-noise robustness, zero-mean Gaussian noise was added to the measured velocity and chamber-pressure signals. When $\sigma_{v}=1.0\;\mathrm{mm}/s$ and $\sigma_{p}=1.0\;\mathrm{bar}$ are used, the mean velocity RMSE over five repeated seeds remains 0.293 mm/s, and the maximum velocity deviation is 1.884±0.015 mm/s. Even when the noise level is increased to $\sigma_{v}=2.0\;\mathrm{mm}/s$ and $\sigma_{p}=2.0\;\mathrm{bar}$, the velocity RMSE is 0.298 mm/s. These results indicate that the observer dynamics and input-increment penalty limit the amplification of measurement noise into the valve-control input.

The online computational burden is mainly associated with the finite-horizon QP solved by the OFMPC. For the selected horizons and constraints, the MATLAB benchmark of the finite-horizon QP implementation gives mean, 95th-percentile, and maximum solution times of 1.188 ms, 2.284 ms, and 9.005 ms, respectively. The average and 95th-percentile computation times occupy 11.9% and 22.8% of the 10 ms control period, and all observed QP calls in this benchmark remain below the control period. A separate delay test shows that increasing the measurement delay to 10 ms and 20 ms leads to maximum velocity deviations of 1.954 mm/s and 2.007 mm/s, respectively, without violating the valve-input constraint. These values indicate that the selected LESO-OFMPC setting satisfies the real-time requirement of the tested control period, while final embedded implementation should still be verified on the target controller hardware.

For a multi-support hydraulic system, the dual-branch configuration used in the experiment can be regarded as a local coupling unit between one target support and its neighboring-disturbance source. For the ith target support, the combined influence of multiple neighboring supports can be represented by aggregated equivalent disturbances acting on the local supply and return nodes:

(96) $$q_{n,s,i}=\sum_{j\in N_{i}}q_{j,s\to i}$$

(97) $$q_{n,r,i}=\sum_{j\in N_{i}}q_{j,r\to i}$$

where Ni denotes the neighboring-support set of the ith target support, $q_{j,s}\to i$ is the equivalent supply-side disturbance transmitted from neighboring support j to target support i, and $q_{j,r\to i}$ is the corresponding return-side disturbance contribution. Since each local controller only requires local target-cylinder displacement, velocity, cap-end pressure, and rod-end pressure feedback, together with the reconstructed boundary variables, the proposed observer–controller structure can be deployed in a distributed manner. The 95th-percentile benchmark time indicates that several local QP updates can be executed sequentially within one 10 ms period on the tested PC/MATLAB environment. Larger support groups should therefore use local or parallel controller execution and explicitly account for communication delay and neighboring-action scheduling.

Overall, the robustness and implementation analyses show that the proposed LESO-OFMPC maintains bounded tracking error and valve-input constraints under moderate parameter mismatch, measurement noise, and implementation delay. The multi-support discussion further indicates that local equivalent disturbance aggregation provides a feasible implementation form for extending the proposed observer–controller structure to support-group hydraulic systems.

## 6. Conclusions

This study addressed the coupled common supply–return pipeline disturbances, disturbance propagation from neighboring support actions, and unmeasurable common pressure boundaries in cooperative pushing of hydraulic supports. An extended-state-observer-enhanced output-feedback model predictive control method was proposed. The main conclusions are as follows.

1. A control-oriented simplified coupled model was established for cooperative pushing of hydraulic supports. The effects of neighboring support start–stop operations and valve closure on the target branch were represented as the supply-side disturbance $q_{n,s}$ and the return-side disturbance $q_{n,r}$. By incorporating the pressure dynamics of the two cylinder chambers, the impedance of the supply main, and the dynamics of the common return main, the model can describe how coupled supply–return disturbances affect the velocity and pressure responses of the target cylinder.
2. A linear extended state observer was developed to reconstruct the common pressure boundaries and neighboring disturbances under limited measurement conditions. Using only the displacement, velocity, and two chamber pressures of the target cylinder, the observer estimated $P_{s}$, $P_{r}$, $q_{n,s}$, and $q_{n,r}$ online. Co-simulation results showed that the observer captured the dominant dynamic variations in the common supply–return pressure boundaries and equivalent neighboring disturbances after valve-closure transients. During t=5.0–6.5 s, the RMSE values for $P_{s}$, $P_{r}$, $q_{n,s}$, and $q_{n,r}$ estimation were 6.1776 bar, 3.6718 bar, 2.6362 L/min, and 0.0066 L/min, respectively.
3. An observer-based output-feedback model predictive controller was constructed by embedding the reconstructed boundary states and disturbance estimates into the prediction model. The controller coordinated displacement tracking, velocity stabilization, pressure-boundary fluctuation suppression, and valve-input smoothing under input-amplitude, input-increment, and output soft constraints. Compared with PID and conventional MPC, LESO-OFMPC reduced the maximum velocity deviation to 1.7484 mm/s, shortened the recovery time within the 1% velocity error band to 0.112 s, and decreased the total variation in the control input to 2.3383. These results indicate that the proposed method can attenuate the transmission of coupled supply–return disturbances to the target-cylinder motion response while improving valve-input smoothness.
4. The dual-branch hydraulic-platform experiment further verified the engineering applicability of the proposed method. After controller intervention, the maximum velocity fluctuation of the target cylinder decreased from 1.0041 mm/s to 0.3011 mm/s, the velocity recovery time was shortened from 0.505 s to 0.038 s, and the peak deviation of the equivalent driving-pressure difference decreased from 0.1933 MPa to 0.1107 MPa. The experimental results demonstrate that the proposed method can mitigate the influence of valve-closure impacts from the disturbance circuit on the motion continuity and driving-pressure stability of the target circuit.

Overall, the proposed method enables online reconstruction of common supply–return boundaries and equivalent neighboring disturbances under limited sensing conditions. By integrating this reconstructed information into output-feedback predictive control, the method reduces the influence of neighboring disturbances on the cooperative pushing process of the target hydraulic cylinder. It therefore provides an implementable solution for unmeasured-state compensation and constrained disturbance suppression in cooperative pushing control of hydraulic support groups.

## Figures and Tables

**Table 1:** Comparison of supply–return disturbance-suppression performance under different control methods.

Index	PID	MPC	DOB-MPC	LESO-OFMPC
Peak velocity after disturbance/mm/s	86.8635	84.1303	82.8058	81.7525
Time of peak velocity/s	5.030	5.025	5.021	5.021
Maximum velocity deviation/mm/s	6.8403	4.1195	2.8058	1.7484
Velocity deviation percentage/%	8.5479	5.1487	3.5073	2.1854
Peak supply-pressure deviation/bar	58.5699	60.3288	61.5785	62.5606
Peak return-pressure deviation/bar	50.6567	46.3046	45.8684	45.7508
Recovery time within 1% velocity error band/s	0.563	0.167	0.161	0.112
Total variation in input, TV_u_	5.9635	2.8808	2.5585	2.3383

**Table 2:** Comparison of experimental indices under the fixed-opening and controller-intervention conditions.

Index	Fixed Opening	Controller Intervention
Peak velocity after disturbance/mm/s	11.0020	10.2995
Time of peak velocity/s	5.029	5.031
Maximum velocity fluctuation/mm/s	1.0041	0.3011
Velocity fluctuation percentage/%	10.0434	3.0111
Recovery time within 2% velocity error band/s	0.505	0.038
Peak deviation of $\Delta P_{e}$/MPa	0.1933	0.1107
Maximum adjustment of target-valve input	0.0000	1.1006
Total variation in target-valve input	0.0000	1.1627

**Table 3:** Robustness test results of the proposed LESO-OFMPC.

Test Item	Setting	Max dev. (mm/s)	RMSE (mm/s)
Nominal response	Baseline	1.875	0.291
Parameter uncertainty	$\tau_{h}-20\%$, $k_{d}+20\%$, $k_{v}+20\%$	2.344	0.343
Sensor noise	$\sigma_{v}=1.0/2.0\;\mathrm{mm}/s$; $\sigma_{p}=1.0/2.0\;\mathrm{bar}$	1.884 ± 0.015	0.293/0.298
Measurement delay	10/20 ms	1.954/2.007	-

Note: $\tau_{h}$ denotes the hydraulic response time constant, $k_{d}$ denotes the equivalent disturbance gain, $k_{v}$ denotes the valve-flow gain, Max dev. denotes the maximum velocity deviation, and RMSE denotes the velocity root mean square error. For the sensor-noise case, $\sigma_{v}$ is in mm/s and $\sigma_{p}$ is in bar.

## Data Availability

The datasets generated and analyzed during the current study, including the co-simulation data and experimental data supporting the figures and tables, are available from the corresponding author upon reasonable request.
